# Electronic Metal-Support Interactions Between Cu_x_O and ZnO for Cu_x_O/ZnO Catalysts With Enhanced CO Oxidation Activity

**DOI:** 10.3389/fchem.2022.912550

**Published:** 2022-05-13

**Authors:** Shuai Lyu, Yuhua Zhang, Zhe Li, Xinyue Liu, Zhenfang Tian, Chengchao Liu, Jinlin Li, Li Wang

**Affiliations:** ^1^ Key Laboratory of Catalysis and Energy Materials Chemistry of Ministry of Education & Hubei Key Laboratory of Catalysis and Materials Science, South-Central Minzu University, Wuhan, China; ^2^ Hubei Key Laboratory of Processing and Application of Catalytic Materials, Huanggang Normal University, Huanggang, China

**Keywords:** metal-support interactions, Cu species, CO oxidation, ZnO, supported catalysts

## Abstract

Metal-support interaction has been one of the main topics of research on supported catalysts all the time. However, many other factors including the particle size, shape and chemical composition can have significant influences on the catalytic performance when considering the role of metal-support interaction. Herein, we have designed a series of Cu_x_O/ZnO catalysts as examples to quantitatively investigate how the metal-support interaction influences the catalytic performance. The electronic metal-support interactions between Cu_x_O and ZnO were regulated successfully without altering the structure of Cu_x_O/ZnO catalyst. Due to the lower work function of ZnO, electrons would transfer from ZnO to CuO, which is favorable for the formation of higher active Cu species. Combined experimental and theoretical calculations revealed that electron-rich interface result from interaction was favorable for the adsorption of oxygen and CO oxidation reaction. Such strategy represents a new direction to boost the catalytic activity of supported catalysts in various applications.

## Introduction

For decades, the Cu/ZnO system has attracted fundamental interest concerning its unique performance in CH_3_OH synthesis, CO_2_ conversion, steam reforming reactions, and oxidation reactions (Behrens et al., 2012; Kuld et al., 2016; Jia et al., 2017; Ye et al., 2018; Jiang et al., 2020). However, the role of the interaction between the two species on the catalytic performance is still not fully understood (Kniep et al., 2004; Liao et al., 2011; Bikaljevic et al., 2019; Zhang et al., 2021). The investigation of this key feature for the performance of Cu/ZnO catalysts has often led to different conclusions. For example, the interface in Cu/ZnO catalysts is crucial for CH_3_OH synthesis via CO_2_ hydrogenation reaction. Although the exact nature of the interfacial sites is still under debate, two possible active sites are generally proposed: Cu-ZnO synergistic sites at the interface and Cu-Zn surface alloy sites (Nakamura et al., 1996; Fujitani and Nakamura, 2000; Kattel et al., 2017a). In other reactions having different activation and reaction conditions, the reconstruction of Cu and Zn species during the reaction is nonnegligible as well. The active species and valence states at the interface are different. Due to the complexity of the catalytic reaction mechanism, there is no unified opinion on the interaction between the two components in Cu/ZnO catalysts (Whittle et al., 2002; Kattel et al., 2017b; Yang et al., 2017).

In fact, revealing the specific structure-activity relationship of supported catalysts is a challenging task because their performance is affected by numerous factors, including the composition, specific surface area, and pore structure of the support, particle size, crystal structure, and morphology of the active metal, and the interaction between the support and the active metal (Li et al., 2018a; Lou et al., 2020a; Lou et al., 2020b; Song et al., 2021). Moreover, these factors generally affect each other. Therefore, to study the effect of one of these parameters while maintaining the other factors constant is not an easy task. For example, to study the pore structure of Al_2_O_3_ on the performance in the Fischer-Tropsch synthesis reaction of a Co/Al_2_O_3_ catalyst, a series of Al_2_O_3_ materials with different pore structures need to be synthesized for the first. However, Al_2_O_3_ supports with different specific surface area or external exposed surface were obtained, which could affect the dispersion degree and size of the Co particles, as well as the interaction between Co having different particle sizes and the Al_2_O_3_ support (Xiong et al., 2005; Liu et al., 2017). Too many influencing factors were introduced during the research, leading to a controversial result. Thus, well-defined catalytic materials and well-designed experiments are essential to obtain a reliable result (Zhang et al., 2014; Yang et al., 2018).

One of the best studied catalytic reactions from a fundamental scientific viewpoint is the CO oxidation reaction, which constitutes an effective pathway to remove exhaust gas (Doherty et al., 2020; Jing et al., 2020). For this reaction, Cu-based materials have been preferentially employed as primary catalysts due to their variable valence state, low temperature reducibility, and low cost (Jernigan and Somorjai, 1994). Although pristine ZnO catalysts lack substantial oxygen vacancy defects to facilitate an efficient CO oxidation, its strong metal-support interaction (SMSI) with expensive noble metals can improve the reaction performance effectively (Liu et al., 2012; Liu et al., 2018). In this context, the combination of ZnO with Cu can be envisaged as a low-cost alternative to catalyze the CO oxidation reaction. However, the role of the interaction between both components in the catalytic performance is still unclear. Thermal decomposition is one of the most effective methods to obtain highly homogeneous nanomaterials (Park et al., 2004). In this paper, a well-defined Cu_x_O/ZnO catalyst was synthesized via a thermal decomposition method. By performing simple efficient treatments, the interaction between Cu_x_O and ZnO was regulated successfully without altering the structure of the catalyst. Using these well-defined catalysts and a series of advanced characterization methods, the role of the interaction between Cu_x_O and ZnO on the CO oxidation performance was investigated thoroughly.

## Experimental

### Chemicals and Reactant Gases

Cu(acac)_2_ (98%; acac = acetylacetonate), benzylamine (98%), and the ZnO support (99%, 100–500 nm) were purchased from Aladdin Chemical Reagent Co., Ltd. Ethanol was purchased from Sinopharm.

CO (99.999% purity), Ar (99.999% purity), O_2_ (99.999% purity), and 10% O_2_/90% Ar gas mixture were purchased from Sichuan Tianyi Science and Technology Co., Ltd.

### Preparation of Catalysts

**Scheme 1:**
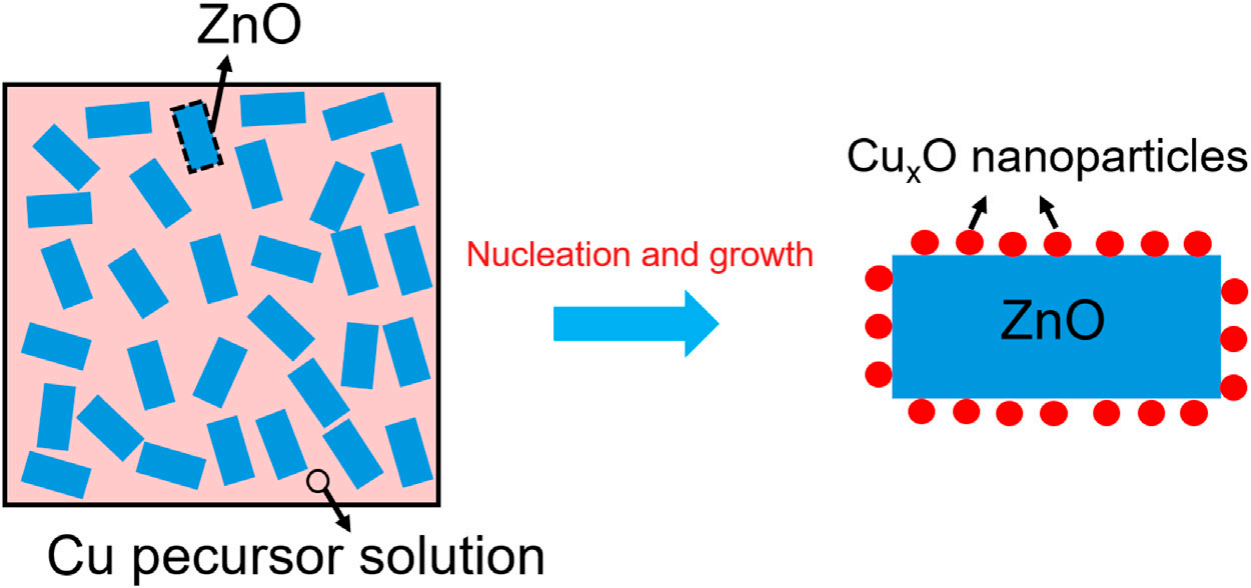
Illustration of the preparation of CuxO/ZnO supported catalysts.

The Cu_x_O/ZnO catalysts were synthesized via a thermal decomposition method, according to which Cu_x_O nanoparticles were allowed to nucleate and grow on the ZnO surface as presented in Scheme 1. In a typical synthesis, 0.626 g of Cu(acac)_2_ (2.4 mmol), 1.8 g of ZnO, and 100 g of benzylamine were suspended in a round-bottomed flask equipped with a reflux condenser. The mixture was ultrasonically dispersed under vigorous stirring for 0.5 h. Subsequently, the obtained solution was heated to 190°C with stirring in an oil bath and refluxed for 2 h. After cooling down to room temperature, the sample was centrifuged and washed with ethanol five times. The obtained catalyst was divided into four equal parts, followed by drying at 200°C for 12, 24, 144, and 324 h in a vacuum drying oven, respectively. The obtained catalyst samples were denoted as Cu_x_O/ZnO-12, Cu_x_O/ZnO-24, Cu_x_O/ZnO-144, and Cu_x_O/ZnO-324 depending on the drying time.

### Materials Characterization

X-ray powder diffraction (XRD) was performed on a Bruker D8 powder diffractometer using Cu Kα radiation (1.5404 Å), operated at 40 kV and 40 mA and a Vantec −1 detector. The size and morphology of the catalysts was investigated using a Tecnai G2 20 S-TWIN transmission electron microscope (TEM) equipped with an energy dispersive X-ray (EDX) spectroscope operated at 200 kV. H2 Temperature-programmed reduction (TPR) was conducted using a Zeton Altamira AMI-300 instrument equipped with a thermal conductivity detector. Prior to the measurement, the catalyst (0.05 g) was flushed with high-purity Ar at 150°C for 1 h and then cooled to 50°C. Then, the temperature was raised to 800°C (ramp rate 10°C min^−1^) by applying a flow of 10% H_2_/Ar at a flow rate of 30 ml min^−1^. Finally, the temperature was held at 800°C for 30 min. X-ray photoelectron spectroscopy (XPS) measurements were performed on a Thermal Electron VG multilab 2000 with an Al K *α* X-ray source under vacuum at 2 × 10^–6^ Pa (the binding energies were corrected using the C 1 s peak at 284.6 eV of the surface adventitious carbon). Electron paramagnetic resonance (EPR) spectra were recorded on a Bruker EMX spectrometer. The EPR experiments were conducted with a center field of 3507.815 G and a frequency of 9.83 GHz using an Elexsys probe head with 15 mg of sample placed in a 4 mm tube. *In situ* diffuse reflectance infrared Fourier transform (DRIFT) spectra of catalysts were collected on a Nicolet Fourier transform infrared spectrometer (NEXUS 470). Before data collection, the catalyst was pretreated with a 10% O_2_/Ar flow rate of 100 ml min^−1^ at 300°C for 1 h to remove absorbed residues and then cooled to 30°C. Pure Ar was introduced to remove O_2_ before CO adsorption experiments.

### DFT Calculations

Density functional theory calculations were performed by PBE (Perdew–Burke–Ernzerhof) method with PAW (projected-augmentation wave) potentials in Vienna ab-initio simulation package program. Plane-wave energy cutoff was set to 400 eV with Gaussian smearing scheme (sigma = 0.05 eV) suitable for semi-conduntor. Total energy and residual atomic force were converged to 10^-4 and 0.03 eV/A.

### Catalytic Test

The CO oxidation reaction was conducted in an atmospheric pressure fixed-bed flow quartz reactor. To prevent temperature gradients, 30 mg of catalyst was diluted with inert SiC powder (0.3 g). The catalyst was pretreated with a 10% O_2_/Ar flow rate of 100 ml min^−1^ at 300 °C for 1 h to remove any absorbed residues and then cooled to room temperature. For the CO oxidation reaction, the reaction temperature was ramped up from 25 to 400°C with a heating rate of 2°C min^−1^. A total flow rate of 100 ml min^−1^ was used, and the space velocity was 200,000 ml (g_cat_ h)^−1^. The outlet gas composition was measured using a gas chromatograph Agilent 7890 BGC equipped with a thermal conductivity detector. The CO conversion was calculated as follows:

$$\mathrm{CO conversion}\left(\%\right)=\frac{CO_{\mathrm{inlet}}-CO_{\mathrm{outlet}}}{CO_{\mathrm{inlet}}}*100\%$$

## Results and Discussion

**FIGURE 1:**
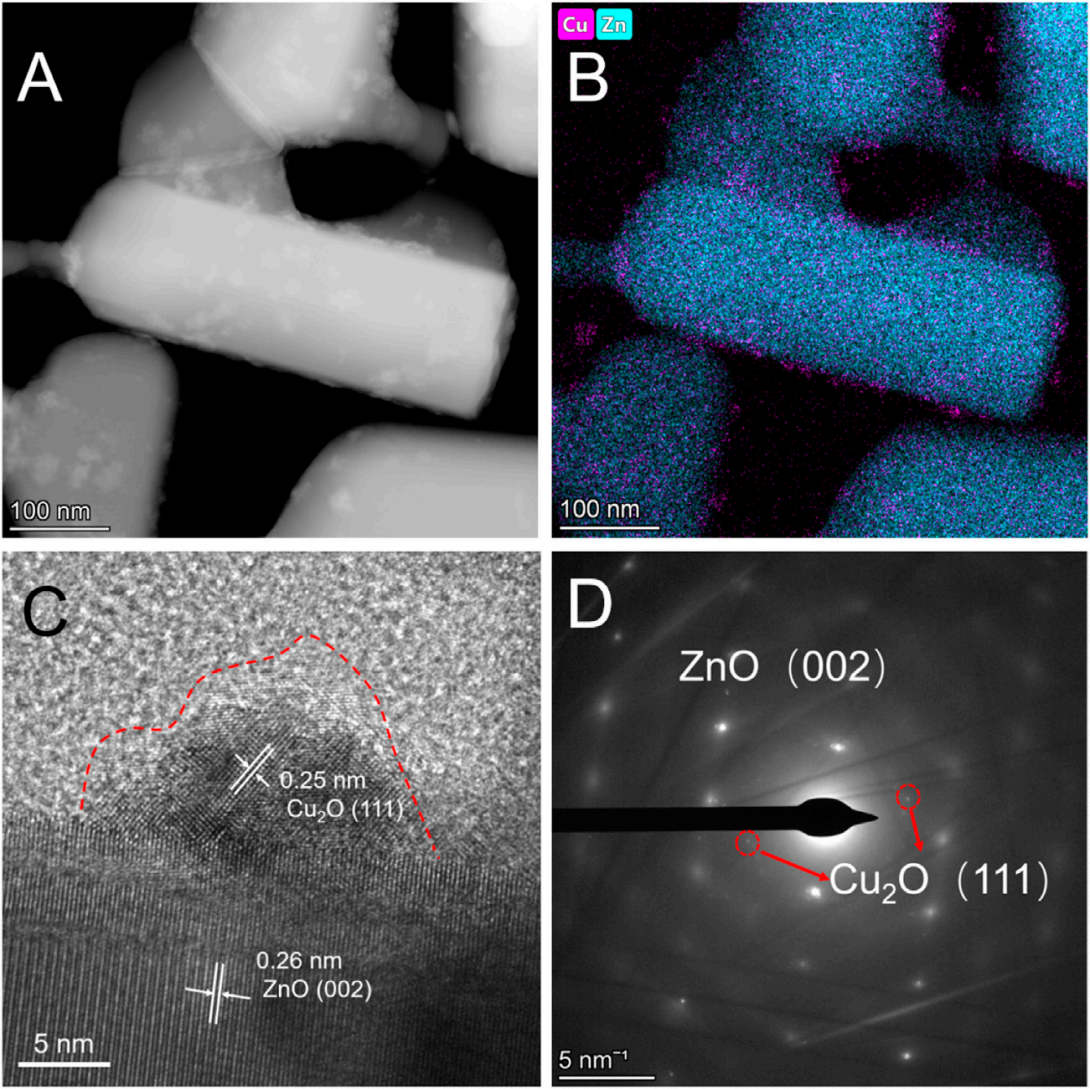
**(A)** High-angle annular dark-field scanning transmission electron microscopy images, **(B)** energy dispersive X-ray mapping, **(C)** high-resolution transmission electron microscopy, and **(D)** selected-area electron diffraction patterns of Cu_x_O/ZnO-12 catalyst.

The Cu_x_O/ZnO-12 catalyst was synthesized *via* a previously reported thermal decomposition approach (Lyu et al., 2019) and dried in a vacuum drying oven at 200°C for 12 h. In the XRD pattern, characteristic diffraction peaks attributed to ZnO (JCPDS # 36-1451) were observed, whereas no peaks corresponding to Cu_x_O appeared (Supplementary Figure S1). This indicates that Cu_x_O particles were well dispersed on the ZnO support. Scanning transmission electron microscopy (STEM) and EDX mapping was used to investigate the morphology and element distribution of the catalysts. The high-angle annular dark-field STEM (HAADF-STEM) images displayed in Figures 1A,B show that Cu_x_O particles with an irregular morphology and broad size distribution were distributed uniformly on the ZnO surface. The selected-area electron diffraction (SEAD) results show that the catalyst was composed of Cu_2_O and ZnO (Figure 1D). Thus, the lattice fringes with a d-spacing of 0.26 nm correspond to the (002) planes of ZnO, and the 0.25 nm lattice fringes can be ascribed to the (111) planes of Cu_2_O. Figure 1C also reveals a distinct oxide layer on the surface of the particles resulting from the formation of CuO after exposure to air and a distinct interface between Cu_x_O and ZnO.

To modify the interaction between the Cu_x_O and ZnO components without affecting the structure of the catalyst, other three Cu_x_O/ZnO samples were prepared using the same procedure described for Cu_x_O/ZnO-12 except that the heating in the vacuum drying oven at 200°C and 0.08 MPa was conducted for 24, 144, and 324 h, respectively. According to Fick’s second law, a concentration gradient due to the different degree of doping with time would cause diffusion of the atoms of the two components into the lattices of each other. The surface reconstruction of the catalyst would be minimized under vacuum. Besides, since the temperature is much lower than the Tammann temperature of Cu_x_O, the agglomeration of Cu_x_O particles would be reduced (Imtiaz et al., 2016). As confirmed by the STEM images of Cu_x_O/ZnO-24, Cu_x_O/ZnO-144, and Cu_x_O/ZnO-324 presented in Supplementary Figure S2–4, the dispersion of Cu_x_O particles was not affected by the treatment time. The change in Cu loading and pore structure was negligible under the present mild treatment conditions.

**FIGURE 2:**
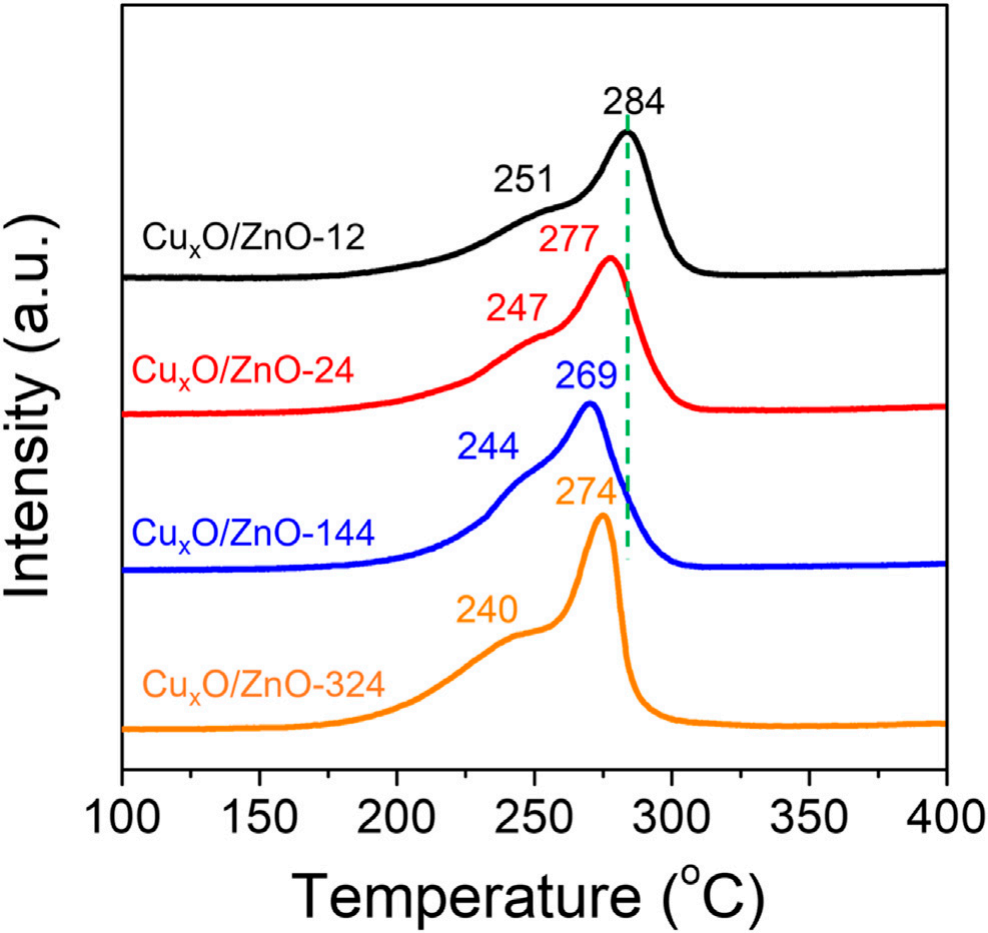
H_2_ Temperature-programmed reduction profiles of the as-synthesized Cu_x_O/ZnO catalysts.

To confirm whether the interaction between the two components in the catalysts was tuned after treatment, the catalysts were characterized by H_2_-TPR. As shown in Figure 2, all the catalysts exhibited two distinct peaks at 220°C–300°C, which are related to the reduction of different Cu_x_O species. The main TPR peak at about 260–300°C can be ascribed to the reduction of bulk Cu_x_O, and a smaller shoulder peak at about 220°C–250°C is attributable to the reduction of Cu_x_O interacting with ZnO, which confirms the existence of a Cu_x_O-ZnO interaction that facilitates the reduction of Cu_x_O species in all the catalysts (Jun et al., 1998; Kniep et al., 2005). Interestingly, the TPR profiles of the whole series of catalysts exhibit similar peak shapes, but the peak position shifts to lower temperature upon increasing the heat treatment time, indicating that the strong interaction with ZnO can promote the reduction of Cu_x_O effectively. This suggests strongly that the Cu_x_O-ZnO interaction was regulated successfully. Thus, a batch of well-defined Cu_x_O/ZnO catalysts with different interactions between components was obtained. It should be pointed out that other structural differences between these catalysts were negligible.

**FIGURE 3:**
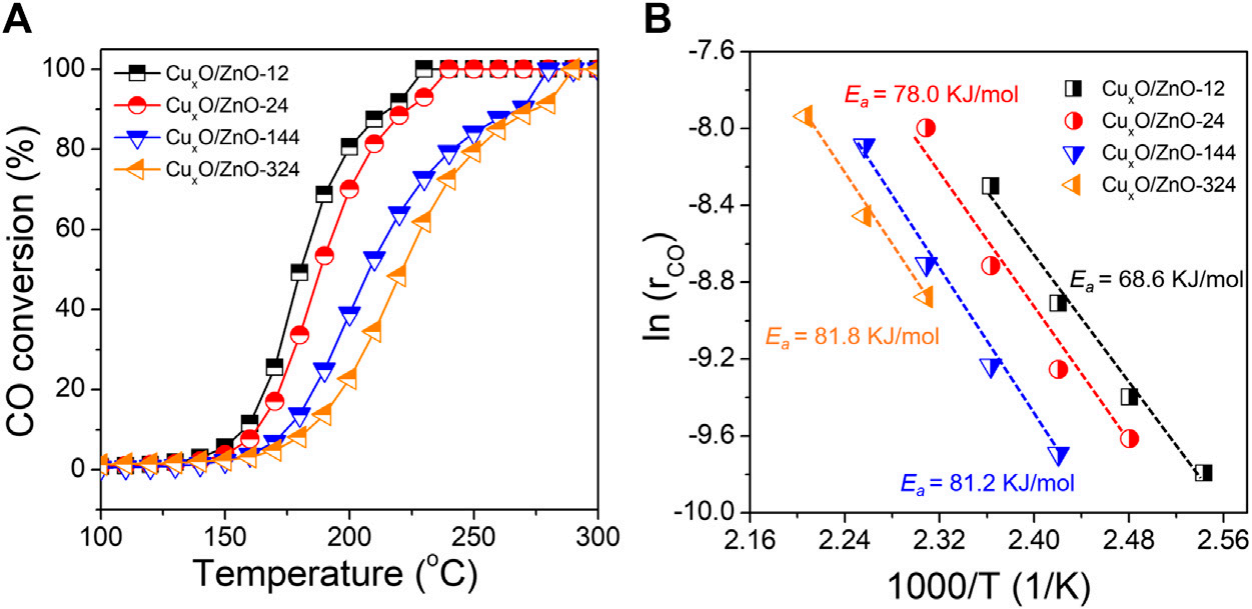
**(A)** CO conversion over the catalyst series as a function of the reaction temperature; **(B)** Arrhenius plots of the different catalysts.

The CO oxidation reaction was conducted on a fix-bed reactor under a gas hourly space velocity of 200,000 ml g_cat_^−1^ h^−1^ (g_cat_, grams of catalyst), which matches standard vehicle exhaust conditions. The light-off curves of the CO conversions are shown in Figure 3. Overall, although the activity of the Cu_x_O/ZnO catalysts was limited, significant differences between the catalysts were observed. Thus, the temperature at which CO conversion is 100% (T_100_) was 230°C for Cu_x_O/ZnO-12, whereas it increased gradually with increasing the heat treatment time. The Arrhenius plots of the CO oxidation rates and the activation energies (*E*_*a*_) are presented in Figure 3B. The Cu_x_O/ZnO-324 sample showed the highest *E*_*a*_ (81.8 kJ/mol), which was much higher than that of Cu_x_O/ZnO-12 (68.6 kJ/mol). The *E*_*a*_ increased monotonously with the drying time.

The different catalytic performance can be ascribed to the structural differences of the catalysts, which are exclusively due to the interaction between the two components, as described above. Although the “metal-support interaction” is a relatively broad concept because the interaction between the two components can be expressed in many forms, it is known to affect the catalytic performance. In a recent review, de Jong et al. (van Deelen et al., 2019) described that several catalyst characteristics such as nanoparticle morphology, charge transfer, interfacial perimeter, chemical composition, and SMSI have a profound effect on the catalytic performance in different reactions. These phenomena are often related to each other and influence the catalytic performance in different degrees, depending on the catalyst and the reaction.

The adhesion energy at the metal-support interface has been reported to affect the shape of the nanoparticles, which has a strong influence on their catalytic performance because different shapes expose certain facets (Bratlie et al., 2007; Xie et al., 2009; Zhou and Li, 2012). Recently, Zhang at al. explored the active sites of a Cu_2_O catalyst in CO oxidation using a series of Cu_2_O particles with regular morphology as model materials, since cubic Cu_2_O particles with different sizes possess different face sites and edge sites (Zhang et al., 2019). Herein, high-resolution TEM (HRTEM) was used to investigate the morphology of the Cu_x_O particles in each catalyst. As shown in Figure 1, the Cu_x_O particles in the Cu_x_O/ZnO-12 catalyst exhibited no special morphology, and no morphological changes were observed upon extending the treatment time (Supplementary Figure S2–4). Therefore, the Cu_x_O morphology is not the reason for the different performance of the catalysts. The STEM result also allows excluding the influence of the interface perimeter between Cu_x_O and ZnO because the size of the Cu_x_O particles is similar in the whole catalyst series. This was also confirmed by the surface element composition data obtained by XPS, according to which the surface Cu content and Cu/ZnO ratio of all the catalysts were almost the same (Supplementary Table S1).

**FIGURE 4:**
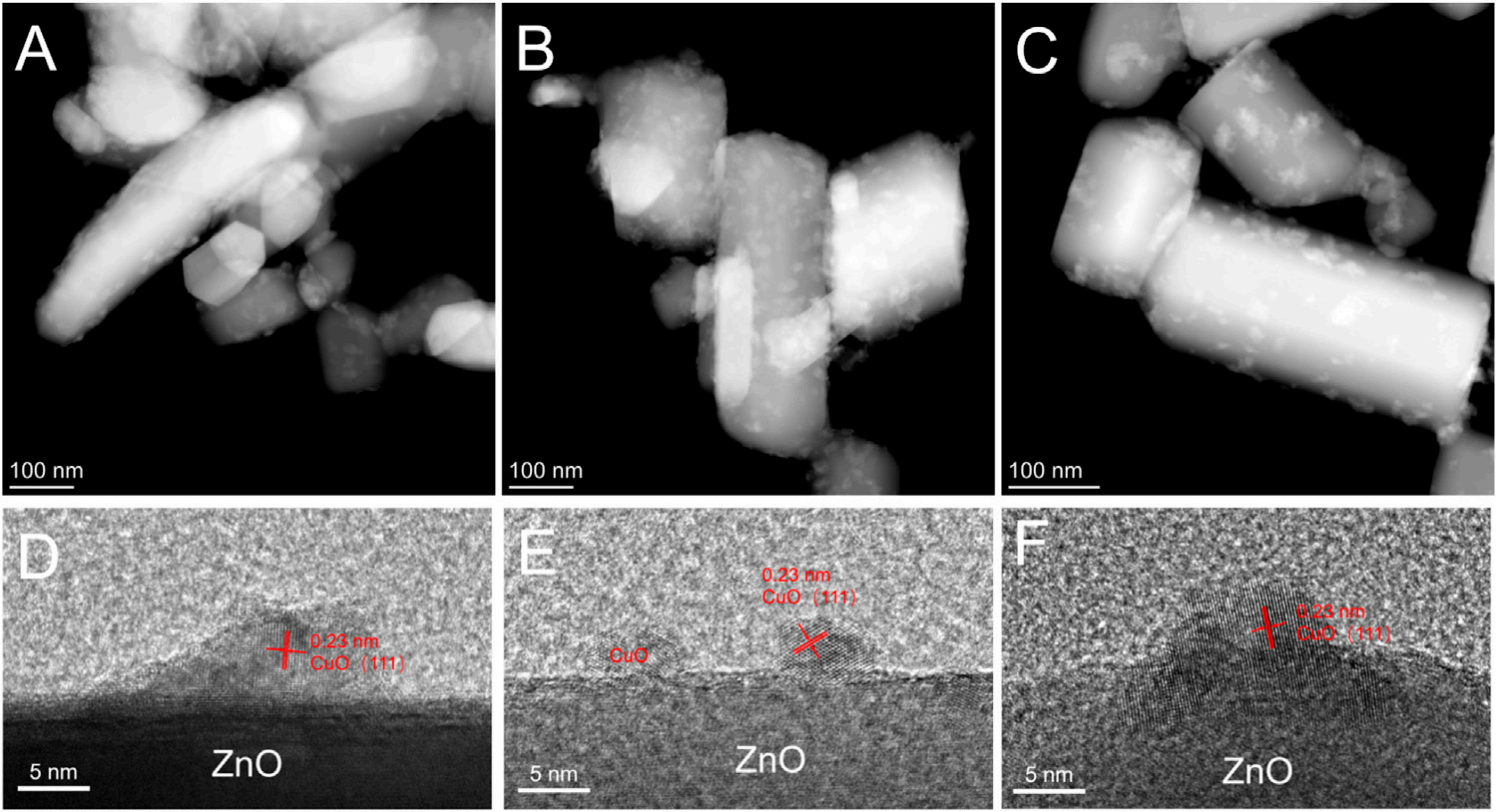
High-angle annular dark-field scanning transmission electron microscopy images of **(A)** used Cu_x_O/ZnO-12 catalyst, **(B)** used Cu_x_O/ZnO-24 catalyst, and **(C)** used Cu_x_O/ZnO-144 catalyst; high-resolution transmission electron microscopy images of **(D)** used Cu_x_O/ZnO-12 catalyst, **(E)** used Cu_x_O/ZnO-24 catalyst, and **(F)** used Cu_x_O/ZnO-144 catalyst.

The term strong metal-support interaction refers to the coverage of metal nanoparticles by suboxides, which are generated from the support under reducing conditions as a result of the surface reconstruction of the catalyst in a specific atmosphere (Liu et al., 2015; Tang et al., 2017; Dong et al., 2020). In the present study, HRTEM was used to examine whether the Cu_x_O particles in the used catalyst were covered by ZnO (Figure 4). After the reaction, the structure of the Cu_x_O-ZnO interface remained virtually unchanged, and no covering was observed. Recently, Luo et al. studied the surface reconstruction of a Cu catalyst during CO oxidation using *in situ* aberration-corrected environmental TEM under O_2_ atmosphere (Luo et al., 2020). However, this nonreducing atmosphere may not be conducive to the formation of SMSI in current case (Yang et al., 2021).

**FIGURE 5:**
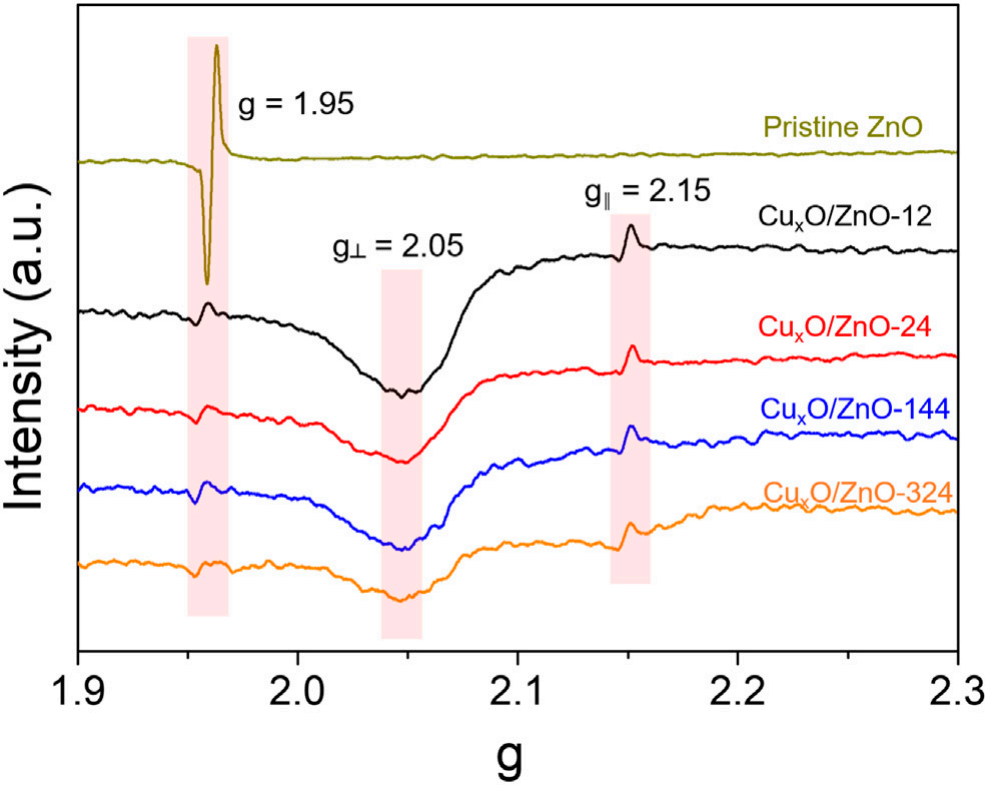
Electron paramagnetic resonance profiles of the as-synthesized Cu_x_O/ZnO catalysts and pristine ZnO support.

A solid-state reaction occurring between metal nanoparticles and the support results in the formation of new phases (Chaika et al., 2020). For Cu/ZnO catalysts, the formation of a Cu-Zn compound induced by hydrogen or a reducing atmosphere often occurs in hydrogenation reactions (Grunwaldt et al., 2000). Meanwhile, the Cu-Zn alloy was reported to be the active center in CO_2_ hydrogenation. In this study, the Cu_x_O/ZnO compound was fabricated according to Fick’s law (Van Milligen et al., 2005), and its formation was confirmed via EPR spectroscopy. As presented in Figure 5, the spectra of all the catalysts exhibited a resonance at g = 1.953, which is attributable to shallow donor centers caused by interstitial Zn and surface O vacancies (Wen et al., 2020). This peak of Cu_x_O/ZnO catalysts was relatively weaker than that of pristine ZnO and decreased slightly with increasing the heat treatment time. Meanwhile, an anisotropic hyperfine structure was observed in all the catalysts. A resonance parameter g_//_ of 2.15 indicates that Cu^2+^ replaced the cation sites of ZnO. Moreover, a broad peak at around g_⊥_ = 2.05 was detected for each sample, which can be ascribed to adsorbed oxygen radicals associated with oxygen vacancies, according to previous studies (Li et al., 2018b). Thus, the EPR results confirm that Cu^2+^ was successfully doped into the ZnO lattice. Notably, the signal assigned to the anisotropic hyperfine structure of Cu^2+^ decreased mildly with increasing the heating time due to the increase of the long-range dipolar interaction between Cu^2+^ ions as the Cu doping amount increased.

**FIGURE 6:**
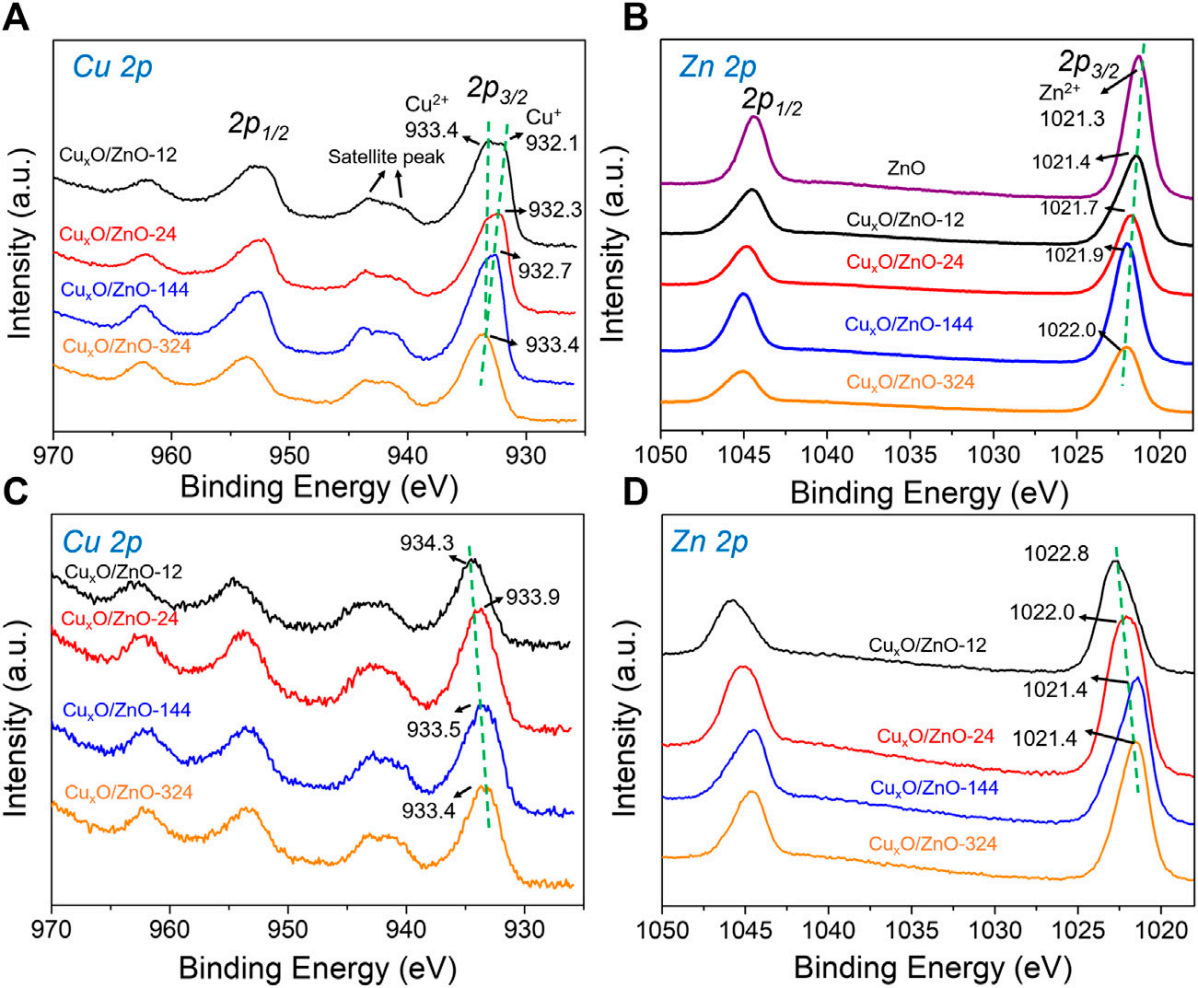
X-ray photoelectron spectra of the catalysts: **(A)** Cu 2p spectra of the fresh catalyst; **(B)** Zn 2p spectra of the fresh catalyst; **(C)** Cu 2p spectra of the used catalyst; **(D)** Zn 2p spectra of the used catalyst.

To investigate the electronic states of the different catalysts, XPS measurements were performed. Figure 6A shows the Cu 2p spectra of the catalysts, in which some differences in the peak shape of Cu 2p_2/3_ can be observed. Thus, the Cu 2p_2/3_ peak for Cu_x_O/ZnO-12, Cu_x_O/ZnO-24, and Cu_x_O/ZnO-144 was clearly split into two peaks: a peak at higher binding energy (933.4 eV) that can be assigned to Cu^2+^ and another peak at ca. 931.9 eV corresponding to Cu^+^. In contrast, only one peak at 933.4 eV was observed in the spectrum of Cu_x_O/ZnO-344 (Svintsitskiy et al., 2013). Interestingly, the Cu^+^ peak shifted gradually toward the high binding energy region as the heat treatment time increased. For Cu_x_O/ZnO-144, the Cu^+^ peak shifted to 932.6 eV. This is a strong indication of the change in the Cu electron state in the catalyst. The electronic state of Zn in the catalyst is also worthy of note.

As shown in Figures 6A,B peak at 1021.4 eV attributable to Zn^2+^ appeared in the spectra of Cu_x_O/ZnO-12 (Zhu et al., 2018). This peak shifted gradually to higher binding energies with increasing the treatment time, indicating that the interaction between the two components changed and the electronic structure of the components was affected simultaneously.

Next, considering that the interaction between the active component and the support of a catalyst is sensitive to the atmosphere (Dong et al., 2018), the electronic property of the used catalysts was investigated by XPS to study the changes in the catalysts during the reaction. An interesting “reverse behavior” of the interaction was observed. As shown in Figure 6C, the surface Cu species of all the catalysts presented characteristic of Cu^2+^ after reaction, in line with the previous studies (Zhang et al., 2019). For the Cu_x_O/ZnO-12 catalyst, the peak corresponding to Cu^2+^ shifted by 0.9 eV to higher binding energy (934.3 eV) compared with that of fresh Cu_x_O/ZnO-12. For Cu_x_O/ZnO-24, the peak shifted by 0.6 eV, for Cu_x_O/ZnO-144 by 0.1 eV, and for Cu_x_O/ZnO-144 by 0 eV. The Zn 2p spectra of the catalysts were also found to change dramatically after the reaction (Figure 6D). Thus, for Cu_x_O/ZnO-12, the binding energy of the Zn 2p_2/3_ peak shifted from 1021.4 to 1022.8 eV. For Cu_x_O/ZnO-24, this shift was 0.7 eV. Meanwhile, the Zn 2p_2/3_ peak shifted to lower binding energy after the reaction for Cu_x_O/ZnO-144 and Cu_x_O/ZnO-324. Taking the Zn 2p_2/3_ peak of pristine ZnO as a reference, the shift value to higher binding energy decreased with increasing the treatment time. In other words, the interaction between Cu_x_O and ZnO in Cu_x_O/ZnO-12 was the strongest among the catalysts after the reaction, whereas that of Cu_x_O/ZnO-324 was the weakest, opposite to the trend for the fresh catalysts.

**FIGURE 7:**
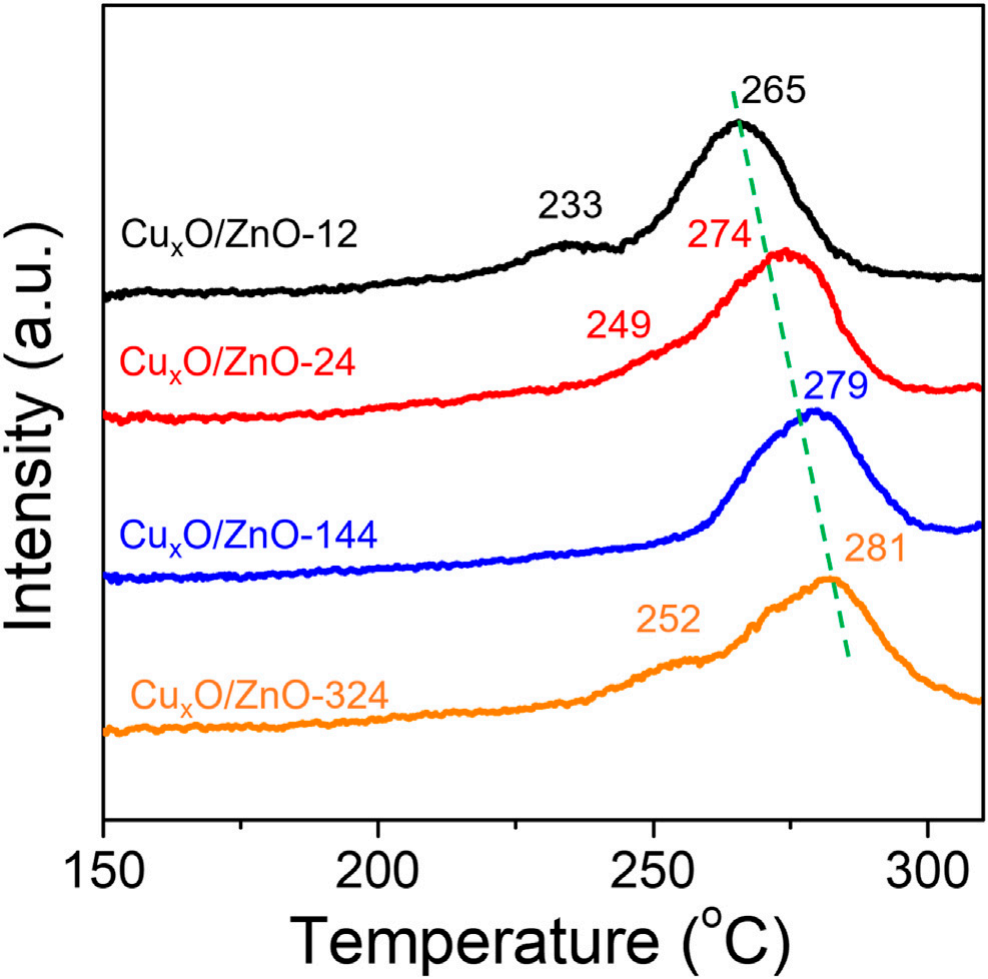
H_2_ temperature-programmed reduction profiles of the used Cu_x_O/ZnO catalysts.

The used catalysts were further characterized by TPR to confirm the change in the interaction for all the catalysts. As shown in Figure 7, the main peak position of used Cu_x_O/ZnO-12 shifted to lower temperature from 284 to 265°C. The reducibility of the catalysts decreased with the treatment time, following an opposite trend to that of the fresh catalysts. Considering that the strong interaction between Cu_x_O and ZnO facilitated the reduction of Cu_x_O, it can be concluded that the interaction between Cu_x_O and ZnO in Cu_x_O/ZnO-12 was the strongest in the series of catalysts after the reaction, which is in line with the XPS results. This suggests a dramatic reconstruction of the catalysts during the reaction. However, the initial strong interactions between Cu_x_O and ZnO impeded the reconstruction process. Therefore, the Cu_x_O/ZnO-12 sample, which had a weaker interaction at the beginning, underwent to a greater extent the reconstruction phenomenon compared with the other catalysts.

**FIGURE 8:**
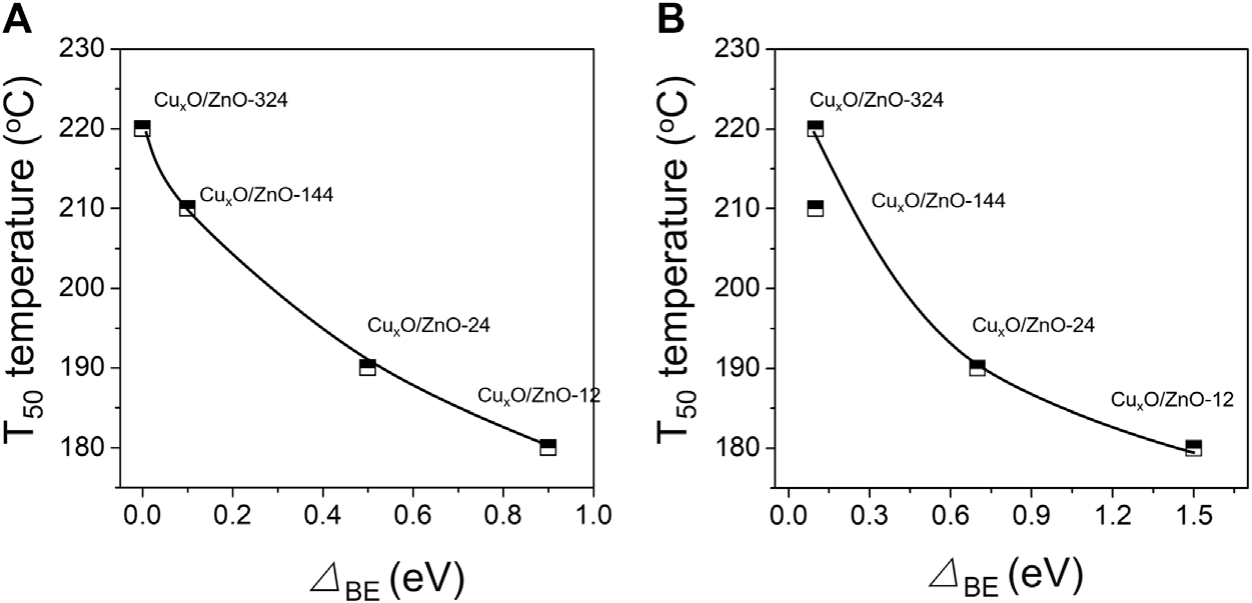
**(A)** Dependence of the T_50_ temperature and the difference between the Cu 2p_2/3_ peak position of the used catalyst and the peak position for Cu^2+^ (933.4 eV); **(B)** dependence of the T_50_ temperature and the difference between the Zn 2p_2/3_ peak position of the used catalyst and the peak position for Zn^2+^ (1021.3 eV).

The electron transfer between the two components was predicted according to the work functions. The work functions calculated by the energy difference between the vacuum and Fermi levels are 5.51, 7.65, 4.57 eV for ZnO(100), CuO(100) and Cu_2_O(111) surfaces respectively, which indicates the shuttling charge transfer between ZnO and CuO/Cu_2_O. In other words, the electrons in Cu^+^ will be transferred to ZnO, which explains the shifting of the Cu^+^ peak in the XPS data toward higher binding energy (Figure 6A). Meanwhile, CuO accepts electrons from ZnO. For the used catalyst, since ZnO is a typical p-typ semiconductor, part of the electrons in the ZnO conduction band are trapped by the adsorbed surface oxygen to form oxygen species, and part of the electrons diffuse into the interface (Yin et al., 2016), which is consistent with the XPS results. After the reaction, the binding energy of the Zn 2p_2/3_ peak of Cu_x_O/ZnO-12 shifted by 1.0 eV compared with that of pristine ZnO, indicating that the strong interaction causes ZnO to release a considerable amount of electrons. The shift value of the Cu 2p_2/3_ and Zn 2p_2/3_ peaks was negatively correlated with the T_50_ temperature (Figure 8). Electron transfer between metal and oxide support is a key step in CO oxidation reaction. In previous studies, White et al. (White et al., 2006) revealed that surface Cu^2+^ was an unlikely candidate for oxidation or O2 dissociation. The active catalyst state was confirmed to be Cu^+^ (in a Cu_2_O lattice) which is either present from the onset or formed by reduction of Cu^2+^ by adsorbed CO. Jernigan et al. (Jernigan and Somorjai, 1994) found that reaction rates for CO oxidation decreased with increasing copper oxidation state (Cu > Cu_2_O > CuO). Huang et al. (Huang and Tsai, 2003) also found that the activity of CuO will be significantly enhanced when non-stoichiometric copper oxides are formed. Thus, it is documented that electrons transfer could contribute to the formation of active Cu species with higher activity, revealing that the strong interaction between Cu_x_O and ZnO is favorable for CO oxidation.

**FIGURE 9:**
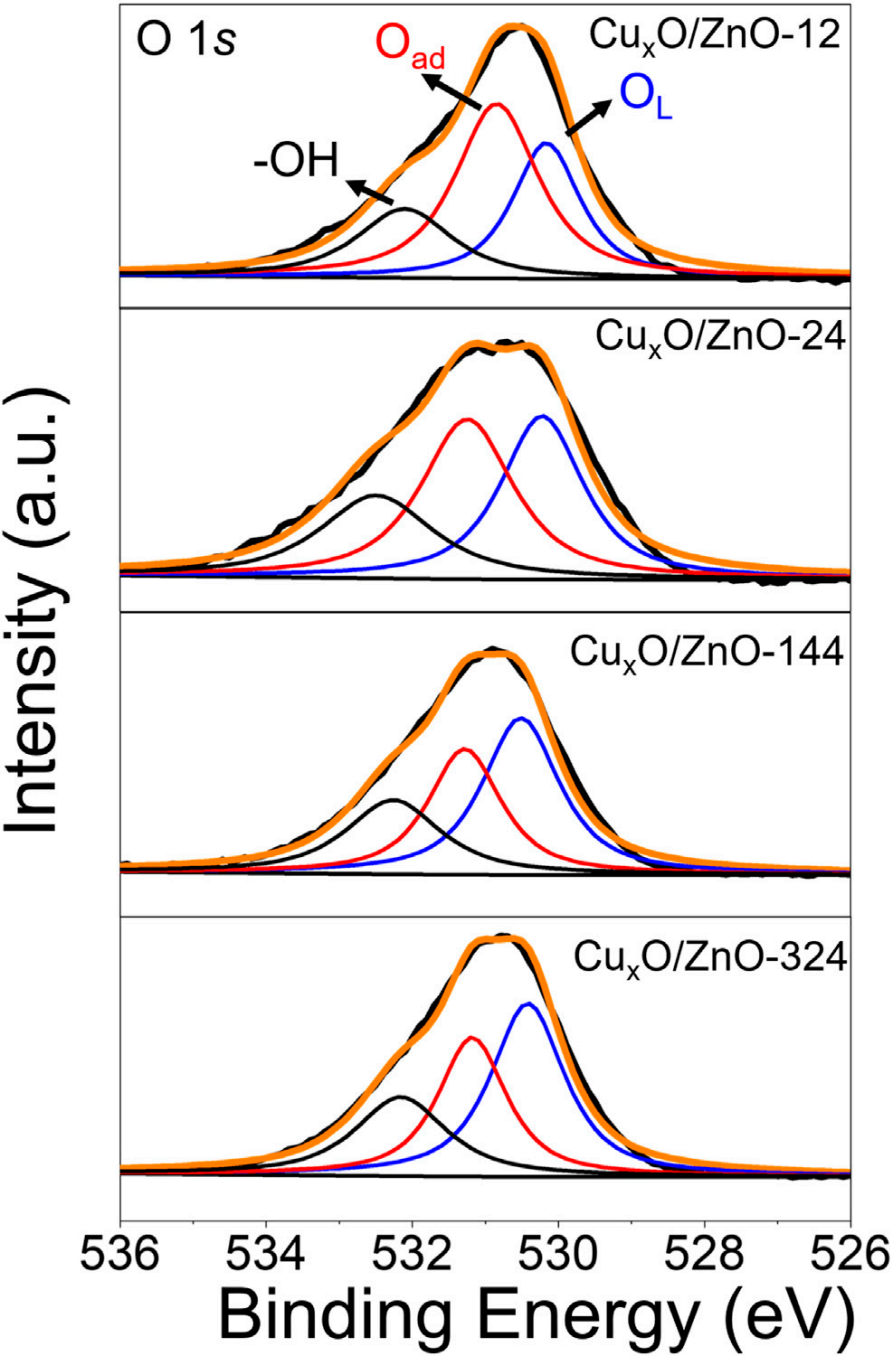
O 1s spectra of the activated Cu_x_O/ZnO. Activated condition: 300 °C under 10% O_2_/Ar for 1 h.

It has been demonstrated that the electron-rich interface would promote the adsorption of oxygen on catalyst (Hayyan et al., 2016; Zeng et al., 2017), which was favorable for CO oxidation reaction. Figure 9 presented the O 1s spectra of activated Cu_x_O/ZnO catalysts. It can be seen that three characteristic peaks could be fitted from the original curve. The peak at around 530.1 eV is assigned to lattice oxygen (O_L_) of catalysts, peak around 531.5 eV is attributed to the adsorbed oxygen (O_ad_) species including O^2−^ and O^2−^ on the surface, the weaker peak at 532.4 eV was the oxygen in hydroxyl groups on the surface. The relative O_ad_ concentration [O_ad_/(O_L_ + O_ad_)] was related to the oxygen storage capacity of catalysts. It can be seen that the activated Cu_x_O/ZnO-12 possess a highest O_ad_ concentration of 61.2% (Supplementary Table S2) and the O_ad_ concentration of Cu_x_O/ZnO catalysts decreased with the treatment time. Thus, the electron-rich interface was favorable for the adsorption of oxygen and CO oxidation reaction.

**FIGURE 10:**
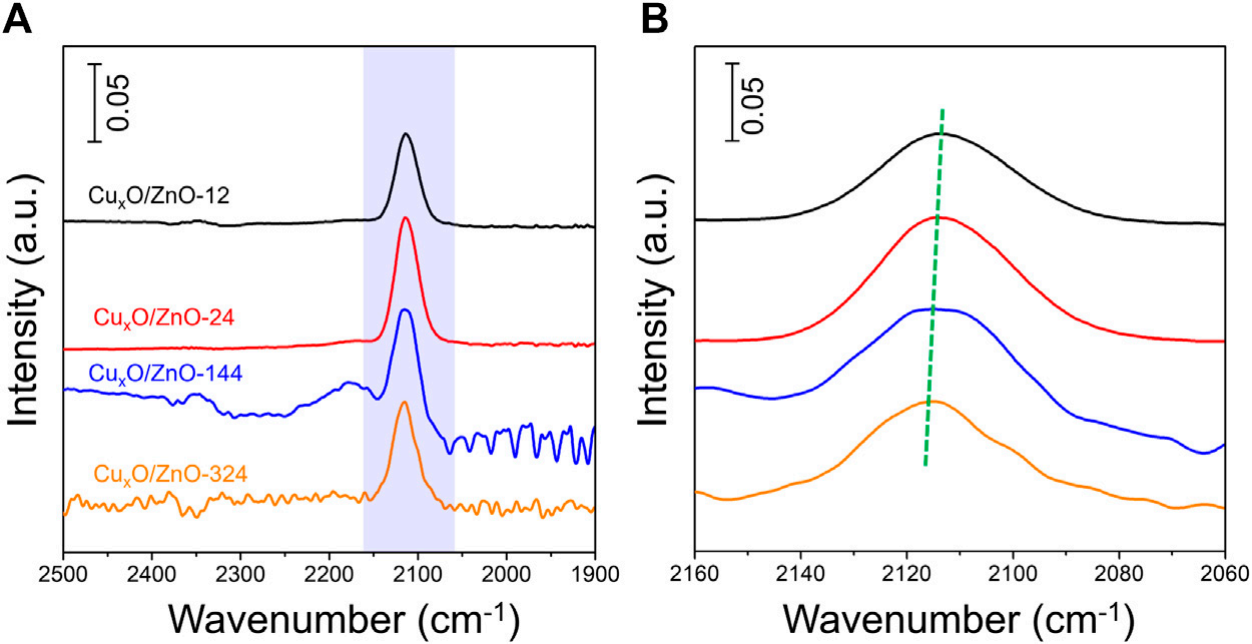
**(A)**: *In-situ* DRIFTS spectra of different Cu_x_O/ZnO catalyst. **(B)**: Spectroscopic data between 2060 and 2160 cm^−1^.

To confirmed further the electronic metal-support interaction between two components. *In situ* DRIFTs experiment was conducted to revealed CO adsorbates behavior on surface of catalysts. Before data collection, the catalyst was pretreated with a 10% O_2_/Ar flow rate of 100 ml min^−1^ at 300 °C for 1 h to remove any absorbed residues and then cooled to 30°C. Supplementary Figure S8 present the spectrum evolution of Cu_x_O/ZnO-12 catalyst at different stage. The peaks appear at 2120 and 2177 cm^−1^ after CO inlet are assigned to the vibrations of gaseous CO. After purging with Ar for 15 min, most gaseous CO in the cell was blew out, a surviving peak at ∼2113 cm^−1^ was assigned to the vibrations of linearly CO species adsorbed on Cu^+^ (Sarkodie et al., 2021). All the catalysts present a distinct CO absorption peak at ∼2113 cm^−1^, as shown in Figure 10A. Moreover, Figure 10B present the detailed absorption peak position of the catalysts, revealing that the absorption peak shift slightly to lower wavenumbers. As established in literature, strong M-C bonds are favored by electron-rich metal centers due to *π* back-bonding, high d-electron density leads to the formation of strong bond between Cu^+^ and CO *via π*-back bonding contributions (Li et al., 2017; Sarkodie et al., 2021). The ZnO support acts as an electronic donor to Cu species would increase the π* back-donation from Cu^+^ to adsorbed CO and hence decrease the carbonyl stretching frequency.

**FIGURE 11:**
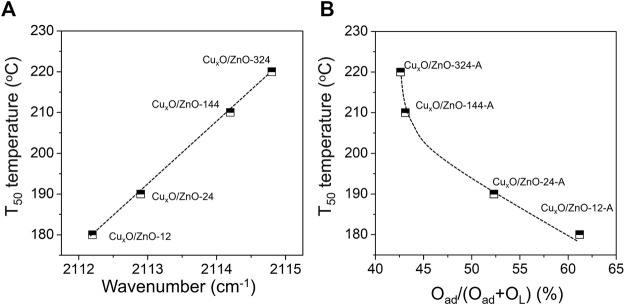
**(A)** Dependence of the T_50_ temperature and the adsorption position of CO molecular; **(B)** dependence of the T_50_ temperature and relative O_ad_ concentration.

Furthermore, *in situ* CO-DRIFTs date and relative O_ad_ concentration data were correlated with activity data of catalyst. As shown in Figure 11A, the T_50_ temperature of catalyst decreased with position of CO adsorption peak, indicating that the activity of Cu_x_O/ZnO catalyst decreases with the decreasing interfacial electron density. From Figure 11B, one can see that the catalytic activity increases with the relative O_ad_ concentration of Cu_x_O/ZnO catalyst.

**FIGURE 12:**
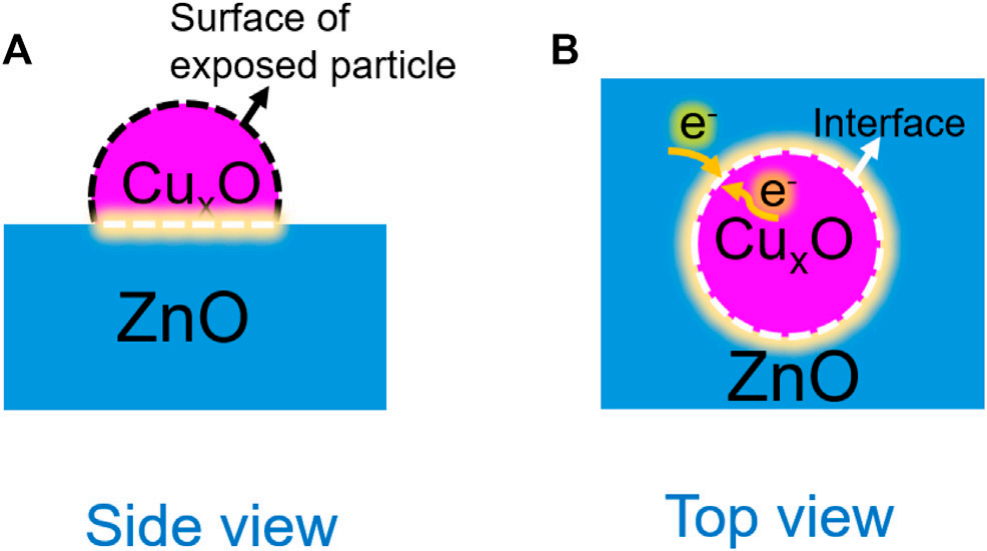
Schematic of the interaction between Cu_x_O and ZnO during the CO oxidation reaction. **(A)**: Side view; **(B)** TOP view.

On the basis of the experimental and theoretical results, the effect of the interaction between Cu_x_O and ZnO on the oxidation performance of CO can be described as depicted in Figure 12. The active sites of the catalysts can be divided into two types: exposed Cu_x_O particles (Figure 12A) and the heterojunction at the interface (Figure 12B). The particle size of the catalysts before and after the reaction was almost unchanged, and the difference in the contribution of exposed Cu_x_O particles on the activity among all the catalysts was negligible. Thus, the electronic structure at the catalyst interface can be considered the main factor affecting the catalytic activity. The strong interaction between the two components promotes the enrichment of electrons at the interface and the activation and dissociation of oxygen molecules. Nevertheless, the catalyst reconstruction during the reaction is an important factor in the formation of a strong catalyst interaction.

## Conclusion

In this study, a well-defined Cu_x_O/ZnO catalyst was synthesized via a typical thermal decomposition method. By adjusting the drying time of the catalyst under vacuum, the Cu_x_O-ZnO interaction was regulated successfully. The interaction between Cu_x_O and ZnO was altered by the reconstruction of the catalyst during the CO oxidation reaction. For catalysts with a weak interaction, the reconstruction process enhanced the interaction between the two components, which was conducive to the electron transfer and the overall activity. In contrast, for the catalysts with a strong interaction, the reconstruction process is blocked. After a restructuring procedure, the strong interaction between Cu_x_O and ZnO is favorable for the CO oxidation reaction.

## Data Availability

The raw data supporting the conclusions of this article will be made available by the authors, without undue reservation.
