# Supplementary material for: Electronic Metal-Support Interactions Between CuxO and ZnO for CuxO/ZnO Catalysts With Enhanced CO Oxidation Activity
Source: Front Chem. 2022 May 13;10:912550. doi: 10.3389/fchem.2022.912550 (PMC9136224; doi:10.3389/fchem.2022.912550)
Supplement: Supplementary file 1 [file DataSheet1.docx]

Supplementary Material

Electronic metal-support interactions between Cu_x_O and ZnO for Cu_x_O/ZnO catalysts with enhanced CO oxidation activity

Shuai Lyu^1^†, Yuhua Zhang^1*^†, Zhe Li,^1^ Xinyue Liu^1^, Zhenfang Tian^2^, Chengchao Liu^1^, Jinlin Li^1^ and Li Wang^1^*

^1^ Key Laboratory of Catalysis and Energy Materials Chemistry of Ministry of Education & Hubei Key Laboratory of Catalysis and Materials Science, South-Central Minzu University, Wuhan 430074, China

^2^ Hubei Key Laboratory of Processing and Application of Catalytic materials, Huanggang Normal University, Huanggang 43800, China.

***Correspondence:**Corresponding Author [poale_zhang@aliyun.com](mailto:poale_zhang@aliyun.com) (Y. Zhang); [li.wang@scuec.edu.cn](mailto:li.wang@scuec.edu.cn) (L. Wang)

**†These authors contributed equally to this work.**


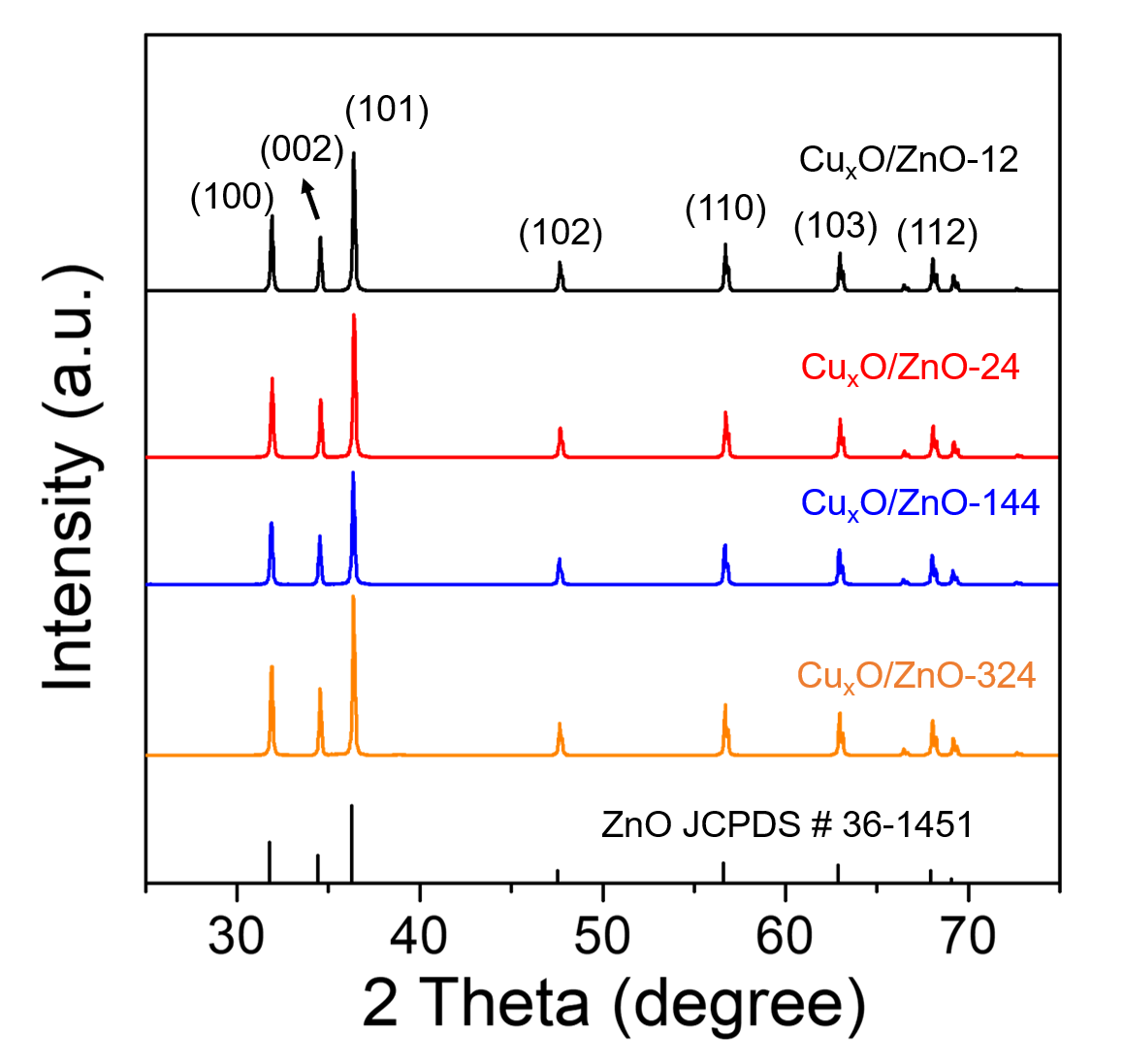


Figure S1 XRD profiles of the as-prepared Cu_x_O/ZnO samples.


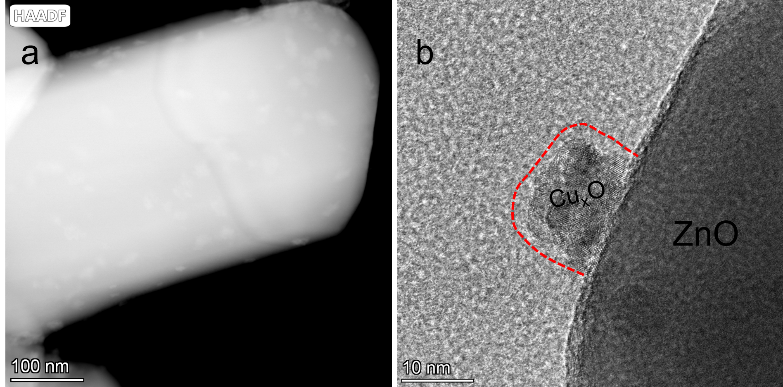


Figure S2 HAADF-STEM and HRTEM image of CuxO/ZnO-24.


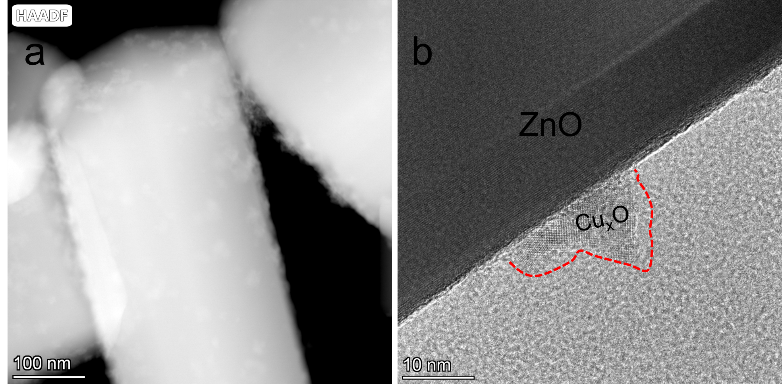


Figure S3 HAADF-STEM and HRTEM image of Cu_x_O/ZnO-144.


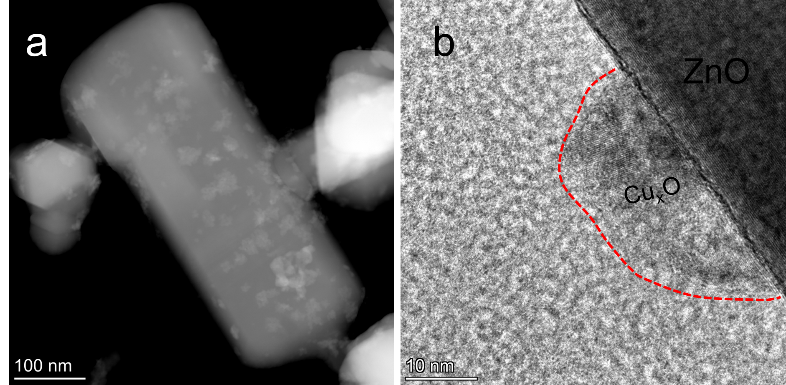


Figure S4 HAADF-STEM and HRTEM image of Cu_x_O/ZnO-324.


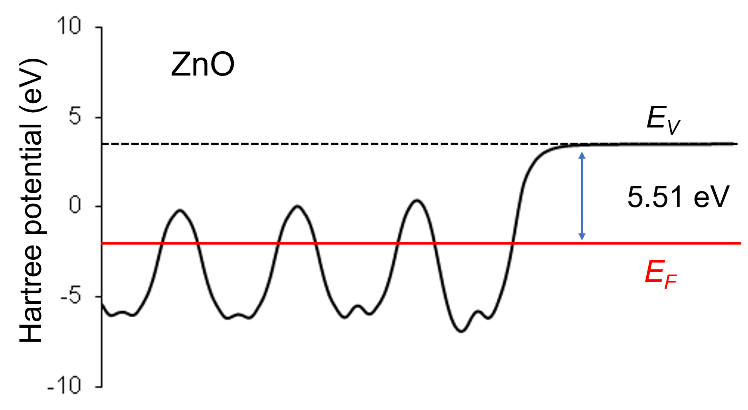


Figure S5 The Hartree potential along c axis of ZnO (100). The red full line and black dashed lines denote the Fermi and vacuum energy levels, respectively.


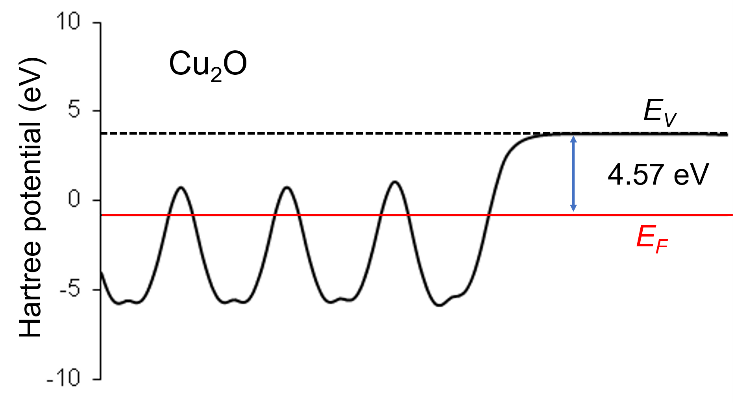


Figure S6 The Hartree potential along c axis of Cu_2_O (111). The red full line and black dashed lines denote the Fermi and vacuum energy levels, respectively.


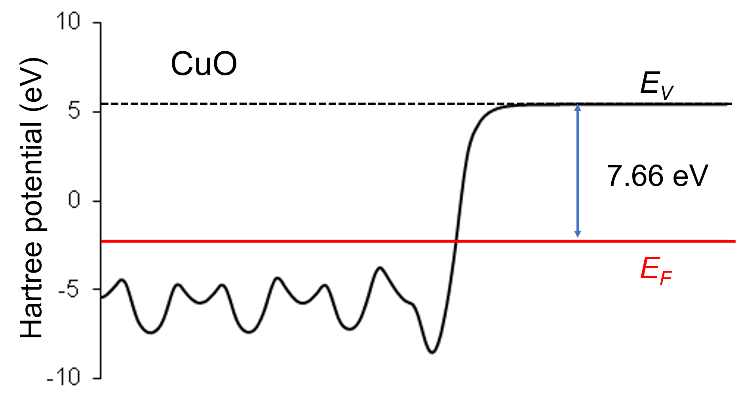


Figure S7 The Hartree potential along c axis of CuO (100). The red full line and black dashed lines denote the Fermi and vacuum energy levels, respectively.


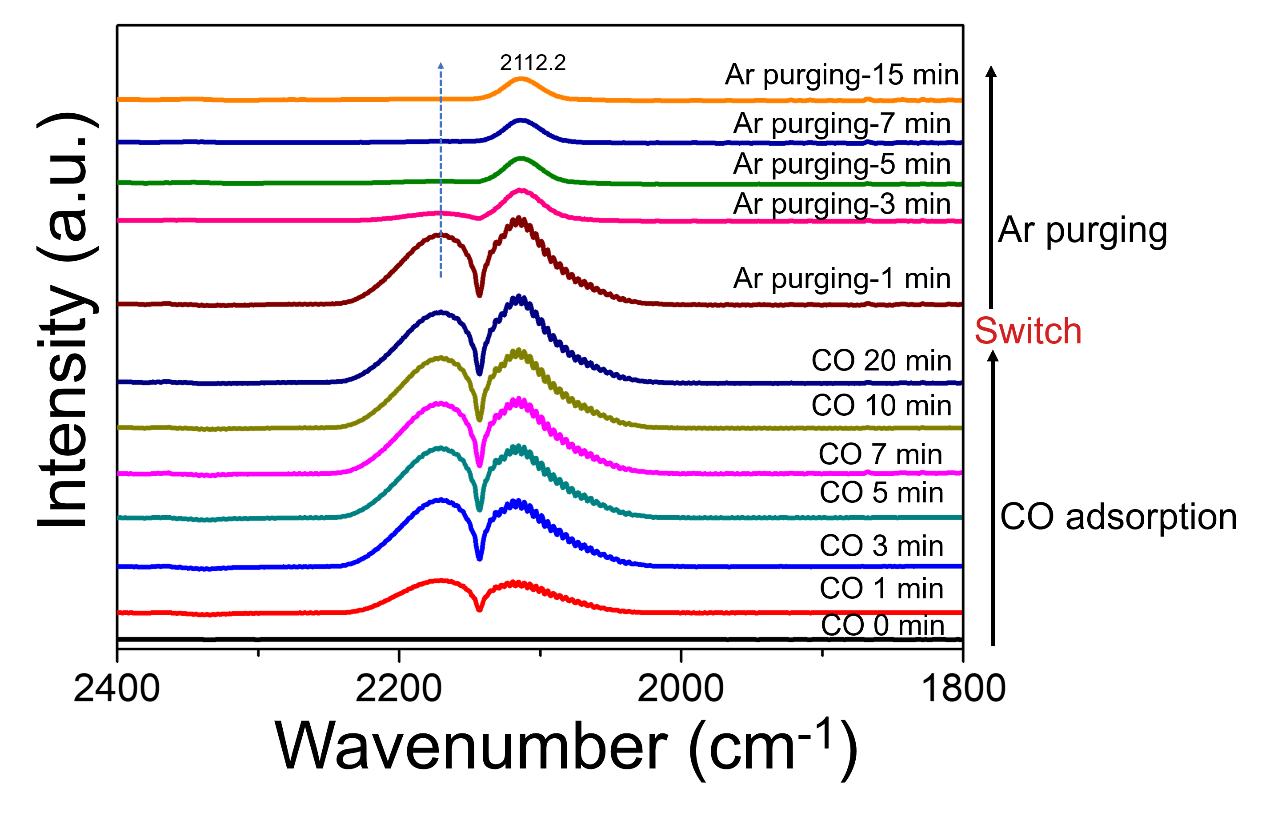


Figure S8. *In-situ* DRIFTS spectra of Cu_x_O/ZnO-12 catalyst under different treatment stage.

Table S1 XPS results for the as-synthesized Cu_x_O/ZnO catalysts.

| Sample | Cu (%) | Zn (%) | O (%) | Cu/Zn |
| --- | --- | --- | --- | --- |
| Cu_x_O/ZnO-12 | 8.6 | 38.9 | 52.5 | 0.22 |
| Cu_x_O/ZnO-24 | 8.5 | 36.7 | 54.8 | 0.23 |
| Cu_x_O/ZnO-144 | 8.7 | 39.6 | 51.7 | 0.22 |
| Cu_x_O/ZnO-324 | 8.6 | 33.3 | 58.1 | 0.25 |

Table S2 O 1s spectra fitting results of as-synthesized Cu_x_O/ZnO catalysts after activated treatment.

| Sample ^a^ | O_OH_ (%) | O_ad_ (%) | O_L_ (%) | O_ad_/(O_ad_+O_L_)(%) |
| --- | --- | --- | --- | --- |
| Cu_x_O/ZnO-12 | 21.8 | 47.9 | 30.3 | 61.2 |
| Cu_x_O/ZnO-24 | 24.9 | 39.3 | 54.8 | 52.3 |
| Cu_x_O/ZnO-144 | 24.9 | 39.5 | 51.7 | 43.1 |
| Cu_x_O/ZnO-324 | 24.4 | 33.3 | 58.1 | 42.6 |

^a^ Activated treatment condition: 300 ℃ under 10% O_2_/Ar for 1 h
